# The long non-coding RNA HOTTIP promotes progression and gemcitabine resistance by regulating HOXA13 in pancreatic cancer

**DOI:** 10.1186/s12967-015-0442-z

**Published:** 2015-03-12

**Authors:** Zhihua Li, Xiaohui Zhao, Yu Zhou, Yimin Liu, Quanbo Zhou, Huilin Ye, YinXue Wang, Jinlong Zeng, Yadong Song, Wenchao Gao, ShangYou Zheng, Baoxiong Zhuang, Huimou Chen, Wenzhu Li, Haigang Li, Haifeng Li, Zhiqiang Fu, Rufu Chen

**Affiliations:** Department of Medical Oncology, Sun Yat-sen Memorial Hospital, Sun Yat-sen University, Guangzhou, China; Department of Pancreaticobiliary Surgery, Hepatobiliary Surgery, Sun Yat-sen Memorial Hospital, Sun Yat-sen University, Guangzhou, China; Department of Radiotherapy, Sun Yat-sen Memorial Hospital, Sun Yat-sen University, Guangzhou, China; Department of Medical Oncology, Zengcheng People’s Hospital, Sun Yat-sen University, Guangzhou, China; Department of Pathology, Sun Yat-sen Memorial Hospital, Sun Yat-sen University, Guangzhou, China; Department of General Surgery, The Second Affiliated Hospital of Sun Yat-sen University, Sun Yat-sen University, 107 Yan-Jiang Xi Road, Guangzhou, 510120 China

**Keywords:** Pancreatic cancer, HOTTIP, Oncogenic, Epithelial-mesenchymal transition, Chemoresistance

## Abstract

**Background:**

The human genome encodes many long non-coding RNAs (lncRNAs). However, their biological functions, molecular mechanisms, and the prognostic value associated with pancreatic ductal adenocarcinoma (PDAC) remain to be elucidated. Here, we identify a fundamental role for the lncRNA HOXA transcript at the distal tip (HOTTIP) in the progression and chemoresistance of PDAC.

**Methods:**

High-throughput microarrays were performed to detect the expression profiles of lncRNAs and messenger RNAs in eight human PDAC tissues and four pancreatic tissues. Quantitative real-time PCR was used to determine the levels of HOTTIP and *HOXA13* transcripts in PDAC cell lines and 90 PDAC samples from patients. HPDE6 cells (immortalized human pancreatic ductal epithelial cells) and corresponding adjacent non-neoplastic tissues were used as controls, respectively. The functions of HOTTIP and HOXA13 in cell proliferation, invasion, and epithelial-mesenchymal transition were evaluated by targeted knockdown *in vitro*. CCK-8 assays, colony formation assays, and xenografts in nude mice were used to investigate whether targeted silencing of HOTTIP could sensitize pancreatic cancer cells to gemcitabine. Immunohistochemistry was performed to investigate the relationship between HOXA13 expression and patient outcome.

**Results:**

Microarray analyses revealed that HOTTIP was one of the most significantly upregulated lncRNAs in PDAC tissues compared with pancreatic tissues. Quantitative PCR further verified that HOTTIP levels were increased in PDAC cell lines and patient samples compared with controls. Functionally, HOTTIP silencing resulted in proliferation arrest by altering cell-cycle progression, and impaired cell invasion by inhibiting epithelial-mesenchymal transition in pancreatic cancer. Additionally, inhibition of HOTTIP potentiated the antitumor effects of gemcitabine *in vitro* and *in vivo*. Furthermore, knockdown of HOXA13 by RNA interference (siHOXA13) revealed that HOTTIP promoted PDAC cell proliferation, invasion, and chemoresistance, at least partly through regulating HOXA13. Immunohistochemistry results revealed that higher HOXA13 expression was correlated with lymph node metastasis, poor histological differentiation, and decreased overall survival in PDAC patients.

**Conclusions:**

As a crucial tumor promoter, HOTTIP promotes cell proliferation, invasion, and chemoresistance by modulating HOXA13. Therefore, the HOTTIP/HOXA13 axis is a potential therapeutic target and molecular biomarker for PDAC.

**Electronic supplementary material:**

The online version of this article (doi:10.1186/s12967-015-0442-z) contains supplementary material, which is available to authorized users.

## Background

Pancreatic ductal adenocarcinoma (PDAC), which accounts for more than 80% of pancreatic cancer cases, is one of the most malignant diseases world-wide. Surgical resection offers the best chance for a possible cure; however, because of the absence of specific symptoms and the lack of early detection, PDAC is usually diagnosed at an advanced stage. Thus, only 10–20% of diagnosed patients present with potentially resectable pancreatic cancer [1,2]. Chemotherapy also plays a critical role in improving symptoms and prolonging overall survival of PDAC patients. However, PDAC is highly resistant to chemotherapy, including gemcitabine treatment, which is generally regarded as the first-line chemotherapy regimen [3]. In the past decades, despite constant efforts to improve the diagnosis and treatment of PDAC, this devastating disease still has a dismal prognosis [4-7]. The roadblocks to major progress are predominantly related to the sophisticated biology and regulatory mechanisms underlying this disease. Hence, a better understanding of the genetic alterations and molecular mechanisms involved in PDAC development and chemoresistance will facilitate the identification of novel diagnostic and therapeutic targets [8].

Studies on human genomes have demonstrated that non-coding RNAs (ncRNAs), which do not encode proteins, account for more than 90% of the transcriptome [9]. The functions of a number of ncRNAs have been characterized, and it is evident that they play crucial roles in the regulation of gene expression, splicing, epigenetic control, chromatin structure, and nuclear transport [10-14]. Among the various classes of ncRNAs, microRNAs (18–200 nucleotides) have been extensively studied, and it has been shown that >1000 microRNAs regulate up to 30% of all protein-encoding genes [13-16]. Additionally, long non-coding RNAs (lncRNAs), defined as non-coding RNAs more than 200 nucleotides in length, are gaining prominence because of their emerging roles in the regulation of critical cellular functions, including the modulation of apoptosis and proliferation, reprogramming of human-induced pluripotent stem cells, tissue differentiation, and as markers of cell fate [17-21]. Importantly, a number of cancer-specific lncRNAs have been identified, which may be employed as novel biomarkers for diagnosis and as therapy targets [22,23].

The HOTTIP lncRNA, located at the 5′ end of the *HOXA* cluster, was recently functionally characterized [24]. Consistent with its genomic position, HOTTIP was significantly expressed in anatomically distal human fibroblasts [24]. The activity of HOTTIP is a result of its interaction with the WDR5/MLL complex, which enhances histone H3 lysine 4 trimethylation to activate the expression of multiple 5′ *HOXA* genes [24]. Recently, it was reported that HOTTIP is a negative prognostic factor in patients with liver cancer, and increased HOTTIP expression was associated with enhanced liver cancer metastasis [25]. In addition, HOTTIP expression is linked to the formation of chemical and ultraviolet radiation-induced skin cancer [26]. However, the underlying role and mechanism of HOTTIP in PDAC remain unknown. The focus of this study was to identify the roles that HOTTIP plays in PDAC, and to uncover the potential mechanisms by which HOTTIP contributes to disease pathogenesis.

In this study, we explored the role of HOTTIP in the regulation of proliferation, invasion, and chemoresistance of pancreatic cancer. We show that targeted silencing of HOTTIP impairs proliferation, invasion, and epithelial-mesenchymal transition ability. Moreover, for the first time, we identify an important role for HOTTIP in gemcitabine chemoresistance in pancreatic cancer cells. Furthermore, we demonstrate that *HOXA13*, which is located in physical contiguity with HOTTIP, is a significant target of HOTTIP, and is involved in the progression of PDAC.

## Methods

### Cell culture

The human pancreatic cancer cell lines, PANC-1, MIA PaCa-2, Capan-2, SW1990, and BxPC-3, were purchased from the American Type Culture Collection and grown in complete growth medium as recommended by the manufacturer, supplemented with 10% FBS and 1% penicillin/streptomycin. HPDE6 cells (immortalized human pancreatic ductal epithelial cells) were obtained from Dr. SN Zhang (Sun Yat-Sen University, Guangdong, China). All cells were cultured in a humidified 5% CO_2_ incubator at 37°C.

### Microarray analysis

Transcriptomic analysis was performed using Arraystar human lncRNA microarrays, V3 (Agilent,Santa Clara, USA), which target 27958 Entrez protein-coding genes and 7419 lncRNAs. Total RNA was extracted and mRNA was purified using the mRNA-ONLY™ Eukaryotic mRNA Isolation Kit (Epicentre). Total RNA was fragmented, labeled (One-Color, Cy3, Agilent), purified, and hybridized with probes in Hybridization Chamber gasket slides (Agilent). The slides were then washed and scanned with an Agilent Microarray Scanner. The raw data were extracted with Agilent Feature Extraction software (Agilent). This software uses the robust multi-array average algorithm to adjust the background signals. Normalized data were obtained after performing the quantile method of intra-microarray normalization and the median method of baseline transformation between the microarrays. Differentially expressed genes with a raw expression level of >400 in more than 4 out of the 12 samples used for profiling were extracted and ordered by p-value. Genes with the highest top 10 p-values were selected for validation. The microarray platform and data were submitted to the Gene Expression Omnibus public database at the National Center for Biotechnology Information (accession number: GSE61166, http://www.ncbi.nlm.nih.gov/geo/query/acc.cgi?acc=gse61166).

### RNA isolation and quantitative real-time Reverse Transcription-PCR (qRT-PCR)

Total RNA was extracted from cells using TRIzol reagent (Invitrogen, San Diego, CA, USA) according to the manufacturer’s instructions. qRT-PCR was performed for *HOTTIP*, *HOXA13*, and EMT markers (*E-cadherin*, *Vimentin*, and *Snai1*), with *GAPDH* as an internal control. RNA was extracted from frozen pancreatic cancer tissues and their corresponding non-neoplastic tissues using TRIzol reagent (Invitrogen) and qRT-PCR was performed for *HOTTIP* and *HOXA13* mRNA using *β-actin* as an internal control. Total RNA was then converted to cDNA by reverse transcription using oligodT primers and SuperScript II reverse transcriptase (Invitrogen). For qRT-PCR, three replicates of each sample were amplified in a 20-μL reaction mixture containing SYBR Green reaction mix (Qiagen,Germany) and 0.5 mM of primer, and analyzed using a Roche Light-Cycler (Roche, Basel, Switzerland). The relative gene expression in cells was determined using the comparative delta-delta CT method (2-∆∆Ct) and the fold change in gene expression of tissues was calculated using the standard ∆∆CT method.

#### HOTTIP and HOXA13 knockdown

The following HOTTIP shRNA and scrambled control shRNA were inserted into the pLVX-tdTomato-Puro lentiviral vector (Open Biosystems, Rockford, IL ). HOTTIP shRNA forward, 5′ -GATCCGCTGCTTTAGAGCCACATATTCAAGAGATATGTGGCTCTAAAGCAGCTTTTTTCTCGAGG-3′ and reverse, 5′-AATTCCTCGAGAAAAAAGCTGCTTTAGAGCCACATATCTCTTGAATATGTGGCTCTAAAGCAGCG-3′. Scrambled control shRNA, forward, 5′-CCGGTTTCTCCGAACGTGTCACGTCTCGAGACGTGACACGTTCGGAGAATTTTTG- 3′ and reverse, 5′ -AATTCAAAAAGTTCTCCGAACGTGTCACGTCTCGAGACGTGACACGTTCGGAGAA- 3′.

shRNA lentivirus was used to generate stable HOTTIP-knockdown cells. Lentiviral particles were produced by transfecting 239 T cells. Viral supernatants were collected 72 h after transfection, and particles were concentrated using a LentiX™ Concentrator overnight at 4°C (Clontech, Mountain View, CA, USA), and aliquots were stored at −80°C. Viral titers of concentrated particles were 1.1 × 10^8^ TU/mL. SW1990 and MIA PaCa-2 cells (5 × 10^5^ cells/well ) were seeded in six-well culture plates and maintained in DMEM with 10% FBS for 24 h prior to infection. For screening, puromycin ( 10 μg/mL ) was added to the medium containing HOTTIP knockdown cells 72 h after infection.

The medium was then replaced every 2 days for 2–3 weeks.

SW1990 and MIA PaCa-2 cells were transfected with siRNAs targeting *HOXA13* mRNA (# SIC002-1NMOL, Sigma Aldrich, St Louis, MO, USA), siHOXA13I sense: 5′-AAUGUAUUUGUGCACCU GCUdTdT-′3/antisense: 3′-dTdTUUACAUAAACACGUGGA-5′; siHOXA13II sense: 5′/5fam/-CCG UCAUGUUUCUCUCUACGAdTdT-3′/antisense: 3′-dTdTGGCAGUACAAAGAGAUGCU-5′) and an off-target negative control (# SIC007MSDS, Sigma Aldrich), using Lipofectamine RNAiMAX (Invitrogen, Grand Island, NY, USA).

### Cell growth and cell-cycle assays

For cell growth assay, SW1990 or MIA PaCa-2 cells with HOTTIP or HOXA13 knockdown were seeded in 96-well plates (1 × 10^3^ cells per well) and pre-incubated at 37°C, 5% CO_2_, in a humidified atmosphere for 0, 24, 48 or 96 h. Counting Kit-8 kit (CCK-8) solution (10 μL, Dojindo Molecular Technologies, Kyushu, Japan) was then added to each well and the plate was incubated for 4 h at 37°C, 5% CO_2_, in a humidified atmosphere. The absorbance was measured at 450 nm using a microplate reader.

For cell-cycle analysis, SW1990 or MIA PaCa-2 cells (5 × 10^4^ cells) with HOTTIP knockdown were collected and washed three times with PBS. Cells were then incubated in propidium iodide (PI) staining solution (RNase A 100 ug/ml and PI 500 ug/ml) for 30 min at 4°C, and cells were analyzed by flow cytometry.

### Boyden chamber cell invasion

Invasion assays were performed using the BD Biocoat Matrigel Invasion Chamber (8 μm; BD Biosciences, San Jose, CA, USA) following the manufacturer’s protocol. After HOTTIP or HOXA13 knockdown, 1 × 10^4^ cells were plated in the upper chamber. The bottom chamber contained medium with 10% FBS to stimulate invasion. After 48 h, the bottom chamber insert was stained with 0.1% crystal violet, and cells were counted by photographing the membrane using a microscope and a × 20 objective lens. Each assay was conducted at least three times.

### Western blot analysis

Cells were washed in PBS and lysed with RIPA buffer (Invitrogen), and a bicinchoninic acid protein assay kit (Pierce, Rockford, IL, USA) was used to calculate the protein concentration of each sample. Equivalent amounts of proteins were separated by SDS-PAGE and transferred to polyvinylidene fluoride membranes for immunoblotting. The membranes were blocked in 5% fat-free milk for 2 h at room temperature, washed three times, then incubated with the following primary antibodies: rabbit anti-human HOXA13 antibody (1:500, #ab26084, Abcam, Cambridge, MA, USA), rabbit anti-human E-cadherin (1:1000, #ab40772, Abcam), rabbit anti-human Vimentin (1:1000; #ab92547, Abcam), rabbit anti-human Snai1 (1:1000, #ab180714, Abcam), and rabbit anti-human GAPDH antibody (1:1000, #ab18162, Abcam). GAPDH was used as a loading control. Horseradish peroxidase-conjugated secondary antibodies (Cell Signaling Technology, Boston, USA) and an ECL chemiluminescence kit (Pierce) were used to detect bound antibody.

### Immunofluorescence (IF) analysis

Following introduction of SW1990 cells with shRNA or siRNA, cells were cultured to confluency on uncoated glass cover slips for 24 h, and fixed in 4% paraformaldehyde at room temperature for 15 min. After washing with PBS, the adherent cell monolayer was permeablilized with 0.1% Triton X-100 in PBS and blocked for 1 h with 10% normal goat or donkey serum in 1% BSA/PBS, followed by overnight incubation at 4°C with primary antibodies: rabbit anti-human E-cadherin (1:100, #ab40772, Abcam), rabbit anti-human Vimentin (1:100; #ab92547, Abcam), rabbit anti-human Snai1 (1:100, #ab180714, Abcam), rabbit anti-human HOXA13 antibody (1:100, #sc-133669, Santa Cruz Biotechnology Inc, Santa Cruz, CA, USA). After washing with PBS, cells were incubated with the Alexa 488-conjugated secondary antibody (#A20181, Molecular Probes, Leiden, The Netherlands) for 1 h. Cells were then washed three times with PBS and mounted in Vectashield containing DAPI (#ab104139, Abcam). The slides were analyzed using a confocal laser scan microscope.

### *In vitro* chemosensitivity assay

SW1990 cells transduceded with shHOTTIP or control shRNA were seeded (5 × 10^3^/well) in 96-well plates. After incubation for 24 h, varying concentrations of gemcitabine (Lilly, Indianapolis, IN, USA) were added to the cells. After 72 h incubation in a humidified atmosphere containing 5% CO_2_, 10 μL CCK-8 reagent was added to each well and cells were incubated for 4 h. The absorbance of each well was determined using an ELISA reader (Wellscan MK3; Labsystems Dragon, Finland) at a wavelength of 450 nm.

For colony formation, SW1990 cells transduced with shHOTTIP or control shRNA were seeded (500 cells/well) in six-well plates overnight and gemcitabine (10 μM) or PBS (100 μL) was added to the cultured cells on the second day. After 14 days, the culture medium was removed, and cells were briefly rinsed with PBS. The cells were then fixed with 4% paraformaldehyde and stained with 0.1% crystal violet, and colonies were counted by visual inspection.

### *In vivo* chemosensitivity assays

All experiments involving animals were conducted according to the institutional guidelines of Guangdong Province and were approved by the institutional guidelines of Guangdong Province and by the Use Committee for Animal Care. BALB/c nude mice (4–6 weeks old) were randomly separated into the following groups (n = 4 mice per group): (a) SW1990-shcontrol, PBS; (b) SW1990-shHOTTIP, PBS; (c) SW1990-shcontrol, gemcitabine; (d) SW1990-shHOTTIP, gemcitabine. Each mouse was inoculated subcutaneously in the dorsal flank with SW1990 cells (3 × 10^6^ cells/mouse) stably transduced with shHOTTIP or control shRNA. When the xenografts reached a mean size of 0.1–0.15 cm^2^ on the 5th day after cell injection, the mice received gemcitabine (120 mg/kg) or PBS (100 μL) via intraperitoneal (i.p.) injection once every 3 days. All groups were treated five times. The tumors were measured every 3–4 days, and tumor volume was calculated using the following formula: volume = (L × W2)/2, where L and W are the longest and shortest diameters, respectively. The mice were sacrificed when the average L of any group reached approximately 1 cm.

### Immunohistochemistry staining and scoring

Paraffin-embedded samples of primary carcinomas were stained for HOXA13. Sections were deparaffinized in xylene and rehydrated in a graded series of ethanol, followed by heat-induced epitope retrieval in citrate buffer (pH = 6.0). Antigen retrieval was performed in 10 mmol/L citrate buffer (pH = 6.0) in a microwave oven for 15 min. The activity of endogenous peroxidases was blocked by the addition of 3% hydrogen peroxide for 10 min at room temperature. Rabbit HOXA13 antibody (#ab26084, Abcam, 1:100) was applied overnight at 4°C, and after washing three times in PBS, sections were immunostained with a goat anti-rabbit secondary antibody (#191866, Abcam, 0.2 ug/ml) for 1 h at 37°C. The slides were incubated with streptavidin-HRP conjugate complex for 45 min at 37°C. After rinsing three times in PBS, the sections were developed with 3,3′-diaminobenzidine. Sections were counterstained with hematoxylin. Sections of skin tissues known to stain positive for HOXA13 were used as positive controls, and normal goat serum and PBS substituting the primary antibody were used as negative controls.

For evaluation and grading of HOXA13 staining results, a scoring criterion previously described by Ohara et al. [27] was used. Briefly, the staining intensity of HOXA13 was graded on a scale of 0–3 (0, none; 1, weak; 2, intermediate; and 3, strong). HOXA13 expression was assessed according to the percentage of staining as follows: 0 points for no staining; 1 point for <25% staining; 2 points for 26–50% staining; 3 points for 51–75% staining; and 4 points for 76–100% staining. The total score was calculated as the product of the scores for the intensity and positive rate of staining. Staining was assessed by two pathologists according to the scoring criteria. Cases with discrepancies were jointly reevaluated until a consensus was reached.

### Patient samples

All samples were obtained from patients undergoing resection of the pancreas at the Sun Yat-Sen Memorial Hospital between 2009 and 2014. Informed consent was obtained from all patients before sample collection. All patients had a clear histological diagnosis. Patient specimens and related clinicopathological data, including complete follow-up, were obtained from the Institute of Pathology and from the Department of Pancreaticobiliary, Sun Yat-Sen Memorial Hospital. All patients in this study met the following criteria: PDAC diagnosis verified by pathological examination, paraffin-embedded tissues were well stored and qualified for serial section, the corresponding tumor tissues and the paired non-tumor tissues were stored in liquid nitrogen immediately following surgical removal, no anticancer treatments given before biopsy collection, and available exhaustive clinicopathologic and follow-up data.

### Statistical analysis

Statistical analyses were performed using SPSS Statistics 16.0 (IBM Chicago, IL, USA). The chi-square test (X^2^ test), Fisher’s exact test for non-parametric variables, and Student’s t test for parametric variables were used (two-tailed). Differences in patient survival were assessed using the Kaplan–Meier method and analyzed using the log-rank test in a univariate analysis. All tests were two-sided, and a p < 0.05 was considered statistically significant.

## Results

### The expression of HOTTIP is increased in PDAC tissues and cell lines

**Figure 1:**
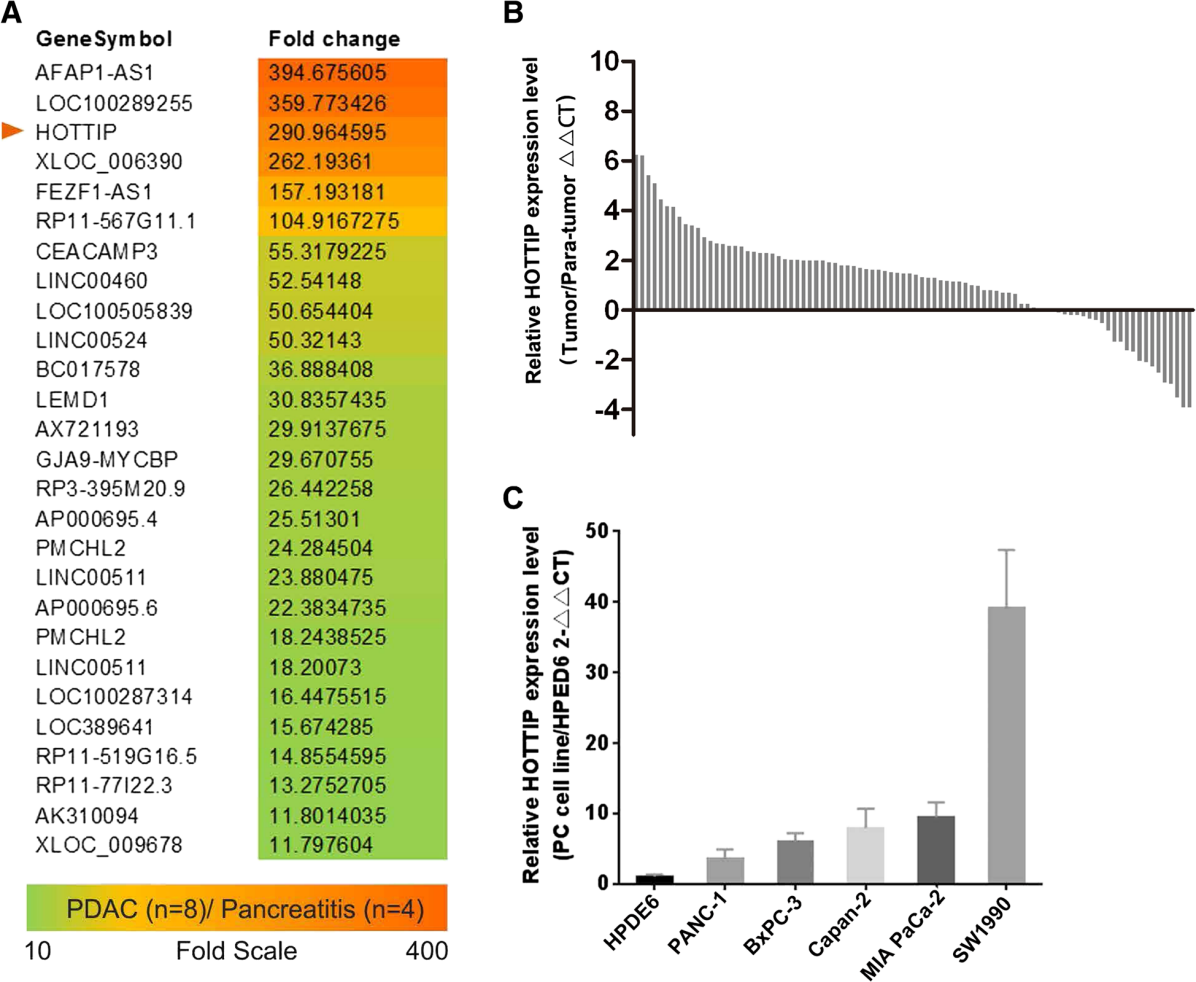
**HOTTIP levels are up-regulated in PDAC tissues and cell lines. (A)** Total RNA from eight cases of pancreatic ductal adenocarcinoma (PDAC) and four cases of chronic pancreatitis were used for microarray analysis. Long noncoding RNAs (lncRNAs) upregulated >10-fold in PDAC tissues (n = 8) compared with chronic pancreatitis tissues (n = 4) are shown. **(B)** HOTTIP expression levels in 90 paired PDAC tissues and corresponding adjacent non-neoplastic tissues was examined via quantitative reverse transcription-polymerase chain reaction (qRT-PCR). *β-actin* was used as internal control. Relative gene expression was determined using the comparative delta-delta CT method, and data are presented as △△Ct. **(C)** HOTTIP expression was evaluated in five pancreatic cancer cell lines compared with immortalized human ductal epithelial cells by qRT-PCR. *HOTTIP* mRNA levels were normalized to *GAPDH*. Data represent the mean ± s.d. from three independent experiments. **p < 0.01, Student’s t-test.

To identify lncRNAs overexpressed in PDAC, we performed gene expression array analysis on eight PDAC tissues and four chronic pancreatitis clinical samples. Twenty seven lncRNAs including HOTTIP, were upregulated more than 10-fold in PDAC compared with chronic pancreatitis tissues (Figure 1A). To further validate these results, we analyzed HOTTIP expression in 90 paired resected samples by qRT-PCR. Compared with adjacent non-tumor tissues, HOTTIP was up-regulated in most PDAC tissues (Figure 1B). We also evaluated the expression of HOTTIP in five pancreatic cancer-derived cell lines (PANC-1, Capan-2, MIA PaCa-2, BxPC-3, and SW1990) and in immortalized human pancreatic ductal epithelial cells (HPDE6) by qRT-PCR. In accordance with the above findings, all pancreatic cancer cell lines exhibited higher levels of HOTTIP compared with the non-tumoral pancreatic cell line, HPDE6, with the highest expression observed in SW1990 cells (Figure 1C).

### HOTTIP regulates cell growth and the cell cycle in PDAC cells

**Figure 2:**
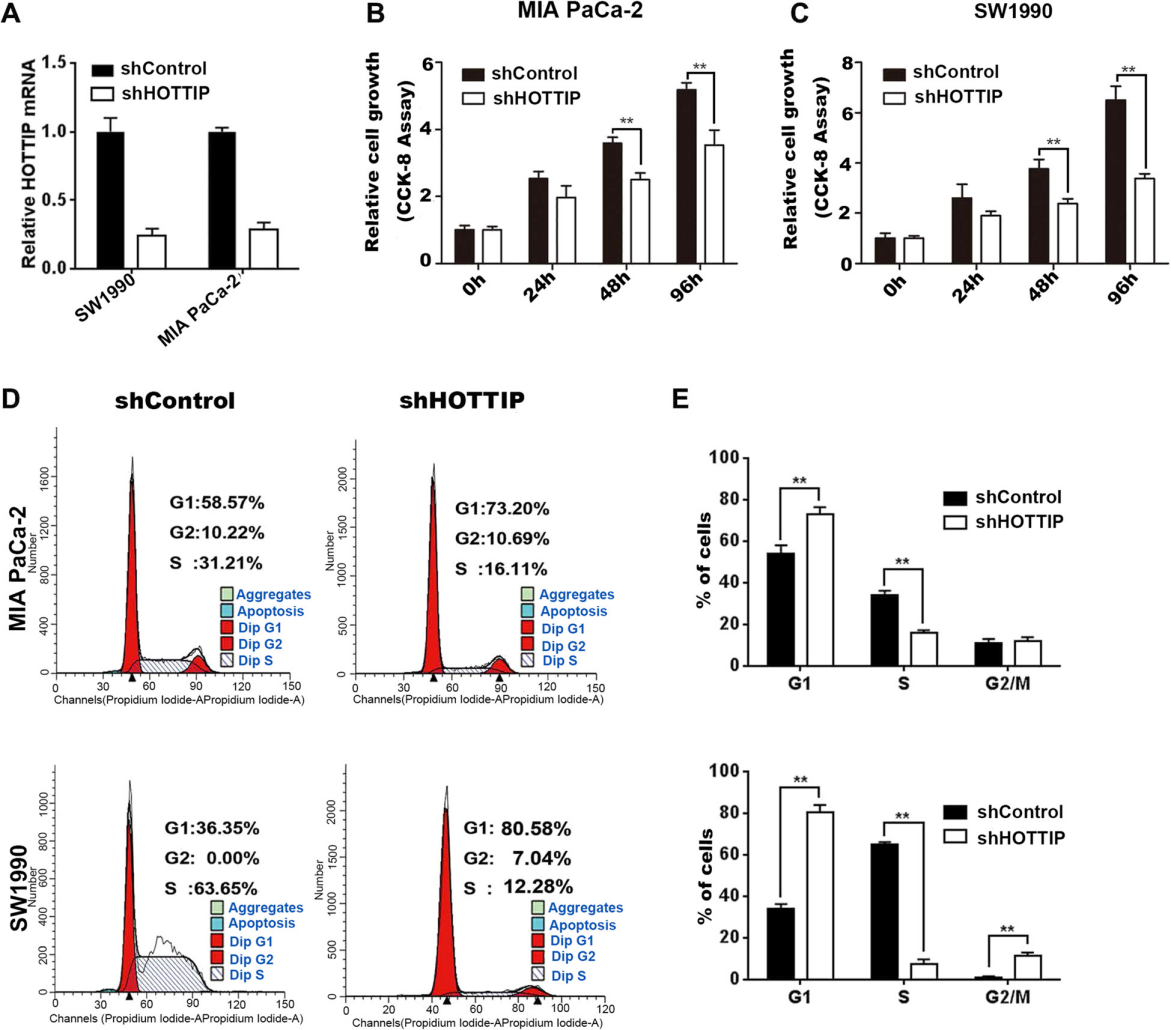
**HOTTIP knockdown inhibits cell growth and cell cycle in PDAC cells. (A)** The efficiency of HOTTIP silencing in short hairpin RNA-stably transduced PDAC cell lines. Relative gene expression was determined using the comparative delta-delta CT method (2^-∆∆Ct^). **(B) (C)** MIA PaCa-2 and SW1990 cells were infected with control shRNA or an shRNA against HOTTIP as indicated. Cell viability was determined at the indicated time points after transfection using CCK-8 assays. **(D) (E)** MIA PaCa-2 and SW1990 cells were tansduced with control shRNA or an shRNA against HOTTIP as indicated. The cell-cycle distribution was assessed following transduction by flow cytometry. Data represent the mean ± s.d. from three independent experiments. **p < 0.01, Student’s t-test.

To investigate the role of HOTTIP in PDAC progression, we first established stable HOTTIP knockdown SW1990 and MIA PaCa-2 cell lines by retrovirus infection (Figure 2A). CCK-8 assays revealed that depletion of HOTTIP reduced cell proliferation compared with shControl in both cell lines (Figure 2B,C). To further investigate the growth inhibition observed following HOTTIP knockdown, we compared the cell-cycle profiles of HOTTIP knockdown cells and controls by flow cytometry. Suppression of HOTTIP led to a decrease in the number of cells in the S-phase and an increase in the percentage of cells in the G0/G1 phase (Figure 2D, 2E).

### HOTTIP regulates PDAC cell invasion and Epithelial-Mesenchymal Transition (EMT)

**Figure 3:**
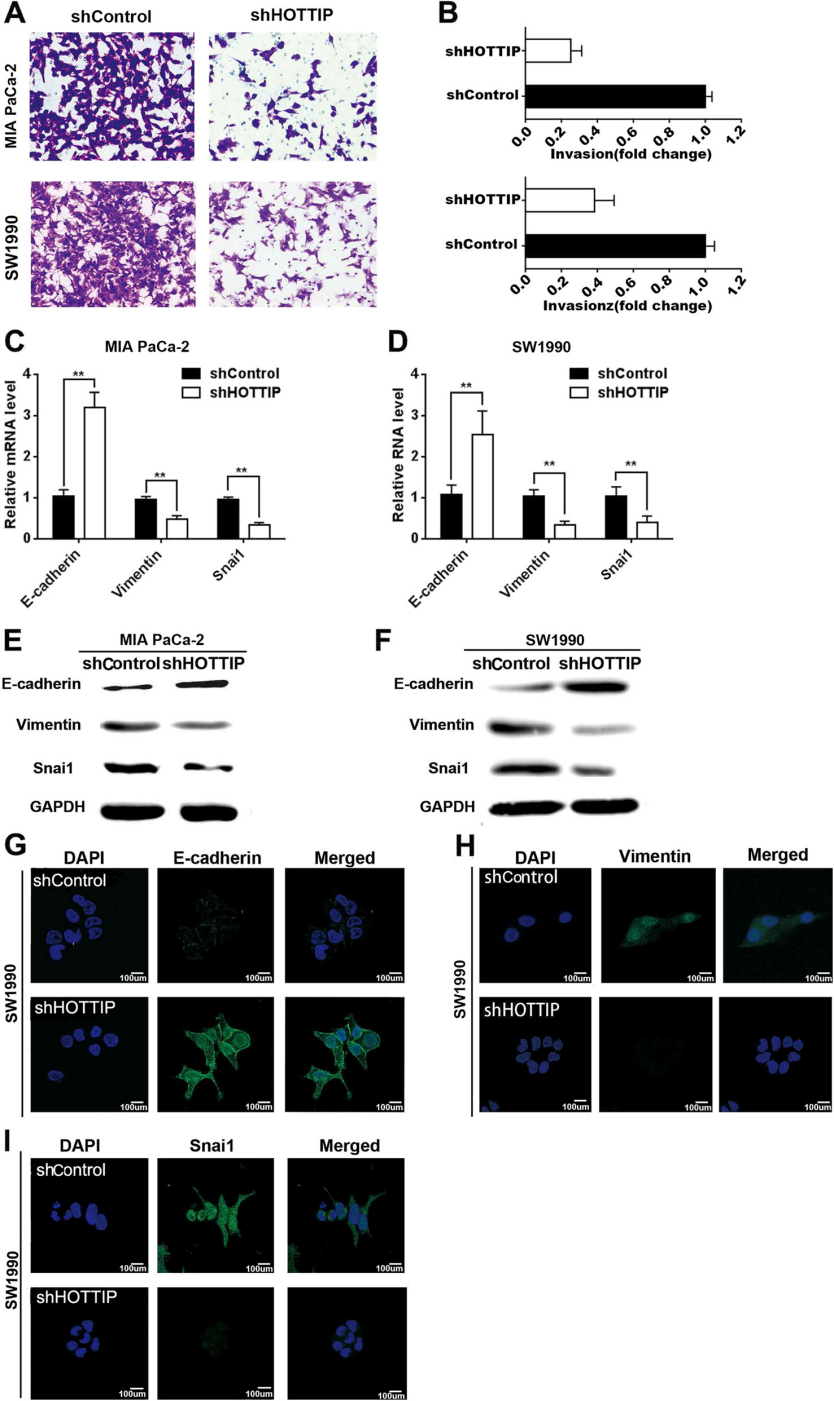
**HOTTIP knockdown inhibits PDAC cell invasion and epithelial-mesenchymal transition** **(EMT). (A)** MIA PaCa-2 and SW1990 cells were transduced with control shRNA or an shRNA against HOTTIP as indicated, and invasion assays were performed. Representative photos of invasive cells on the membrane are shown. **(B)** The effect of HOTTIP knockdown on the invasion ability of MIA PaCa-2 cells and SW1990 cells was quantified by counting the number invasive cells. **(C) (D)** The effect of HOTTIP knockdown on expression of *E-cadherin*, *Vimentin*, and *Snail 1* was evaluated by qRT-PCR. **(E) (F)** The effect of HOTTIP-knockdown on E-cadherin, Vimentin, and Snail 1 protein levels was confirmed by western blotting. **(G) (H) (I)** Immunofluorescence staining for the EMT makers in SW1990 cells. Data represent the mean ± s.d. from three independent experiments. **p < 0.01, Student’s t-test.

We next investigated the effect of HOTTIP on PDAC cell invasion. We first examined the effect of HOTTIP stable knockdown on SW1990 and MIA PaCa-2 cell invasion using transwell assays. HOTTIP knockdown dramatically reduced the invasion of SW1990 and MIA PaCa-2 cells (Figure 3A). Quantification of invading cells revealed a significant decrease in the number of invading cells for both cell lines after HOTTIP knockdown (Figure 3B). Because EMT is vital for PDAC cell invasion, we next examined whether silencing HOTTIP expression inhibited mesenchymal features. As expected, HOTTIP knockdown decreased the expression of Vimentin and Snai1 and increased E-cadherin expression, at both the mRNA (Figure 3C,D) and protein (Figure 3E,F) levels. Therefore, inhibition of HOTTIP in SW1990 and MIA PaCa-2 cells changed the cell morphology from a mesenchymal to a more epithelial phenotype (Figure 3G,H,I).

### HOTTIP knockdown enhances the chemosensitivity of human pancreatic cancer cells to gemcitabine *in vitro* and *in vivo*

**Figure 4:**
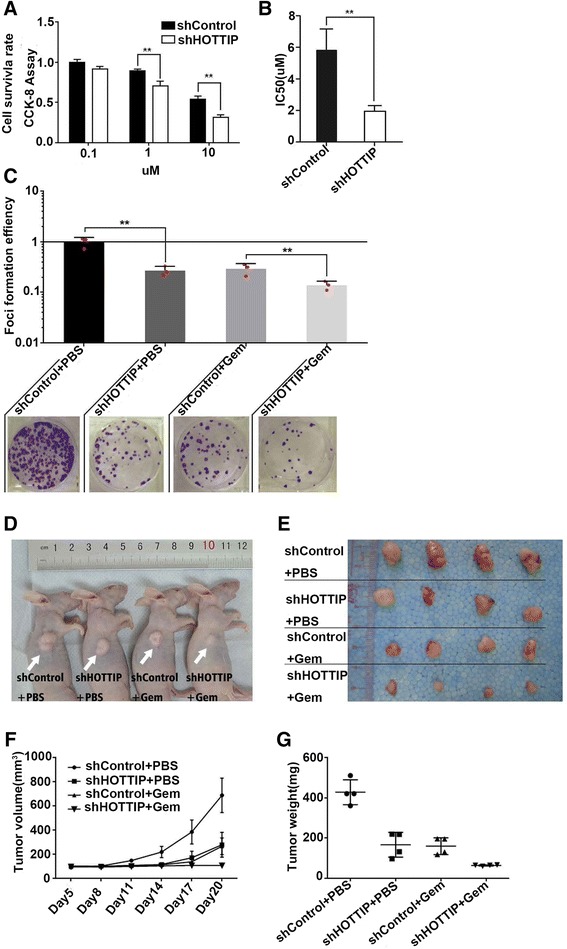
**HOTTIP knockdown enhances the chemosensitivity of human pancreatic cancer cells to gemcitabine** ***in vitro*** **and** ***in vivo.*** **(A)** SW1990 cells were infected with control shRNA or an shRNA against HOTTIP as indicated, and then treated with gemcitabine (0.1, 1, or 10 μM) for 72 h. Cell survival rates were then measured using CCK-8 assays. The values are presented as the means ± s.d. **P < 0.01. **(B)** The effect of HOTTIP suppression on the IC50 of SW1990 cells was calculated. **(C)** SW1990 cells were infected with control shRNA or shRNA against HOTTIP as indicated and then treated with gemcitabine (10 μM) or PBS (100 μL) for 14 days. Colony formation on plastic was then assessed. Representative plates are shown. The number of colonies on each plate was calculated using Image J. The first group was set as the control group, and served as the baseline for colony number normalization. **(D)** SW1990 cells were transduced with control shRNA or shRNA against HOTTIP as indicated. Cells (3 × 10^5^) were then subcutaneously injected into mice, and gemcitabine or PBS were injected intraperitoneally once every 3 days for five cycles. Representative images of tumor-bearing mice are shown. **(E)** Images of tumors from all mice in each group. **(F)** Tumor volumes were measured on the indicated days. **(G)** Tumor weights were determined. *In vitro* data are represented as the mean ± s.d. from three independent experiments. **p < 0.01, Student’s t-test.

Innate or acquired resistance to chemotherapy is a hallmark of PDAC. Considering the effect of HOTTIP on cell proliferation, cell cycle, and EMT identified above, we further tested whether down-regulation of HOTTIP impaired the resistance of PDAC cells to gemcitabine. SW1990 cells stably knocked down for HOTTIP expression (SW1990-shHOTTIP) or infected with control shRNA (SW1990-shcontrol) were exposed to 0.1, 1, and 10 μM gemcitabine for 72 h, and IC50 values were calculated using the CCK-8 cytotoxicity assay (Figure 4A,B). Compared with SW1990 control cells, SW1990-shHOTTIP knockdown cells exhibited much slower growth (Figure 4A) and a lower IC50 for gemcitabine (1.956 ± 0.353 μM vs 5.808 ± 1.361 μM) (Figure 4B). Colony formation was next examined using SW1990 cells stably transduced with shHOTTIP or control shRNA, following exposure to either gemcitabine or PBS for 14 days. Colony growth was significantly inhibited after treatment with gemcitabine (n = 4) or following HOTTIP-knockdown, compared with control. Moreover, combined gemcitabine treatment and HOTTIP knockdown led to a significant reduction in colony formation compared with either treatment alone or the control (Figure 4C).

The ability of HOTTIP to confer chemoresistance in PDAC was further examined using an *in vivo* tumor model. As shown in Figure 4D and E, tumor growth was inhibited in nude mice treated with gemcitabine alone (n = 4) or following HOTTIP-knockdown (n = 4) compared with the controls (n = 4). Tumor growth rate was most significantly inhibited in mice following combined gemcitabine treatment and HOTTIP knockdown. At 21 days, the mean tumor volume for the gemcitabine group and the HOTTIP-knockdown group was markedly smaller than that of the control group (265.0 ± 34.31 mm^3^, 277.9 ± 51.42 mm^3^, and 687 ± 71.26 mm^3^, respectively) (Figure 4F). Furthermore, combined treatment with gemcitabine and HOTTIP knockdown (n = 4) led to an even further reduction in tumor volume (107 ± 34.31 mm^3^). As expected, the tumor weight at the end of the gemcitabine treatment period showed a similar trend (Figure 4G).

#### HOTTIP knockdown leads to decreased HOXA13 expression

**Figure 5:**
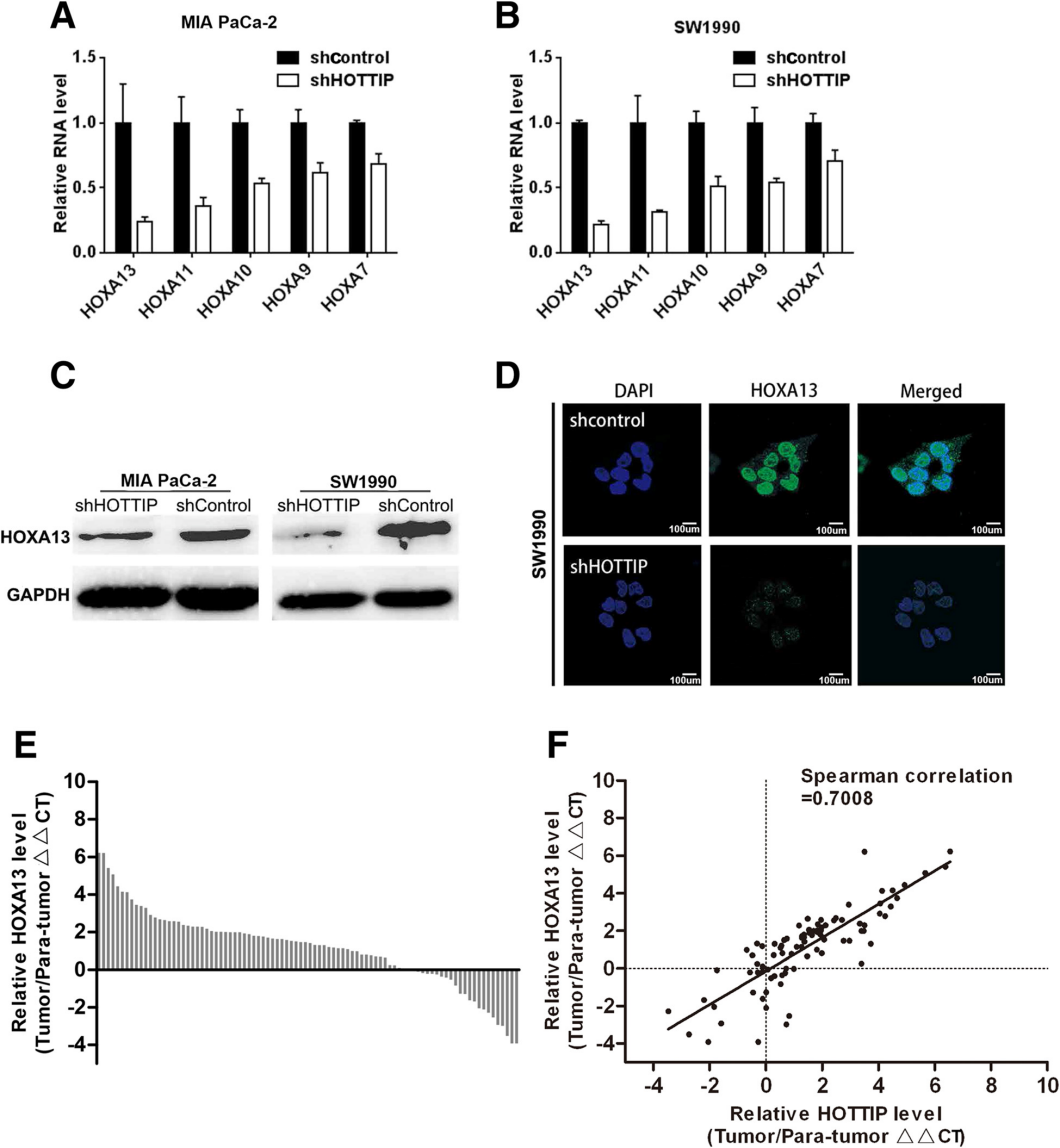
**HOTTIP knockdown inhibits HOXA13 expression. (A)** **(B)** MIA PaCa-2 and SW1990 cells were transduced with control shRNA or shRNA against HOTTIP as indicated. Knockdown of HOTTIP abrogated the expression of *HOXA* genes in the PDAC cell lines, with the strongest inhibition observed for *HOXA13*. **(C)** The effect of HOTTIP knockdown on HOXA13 protein levels was evaluated by western blotting. **(D)** The effect of HOTTIP on HOXA13 expression was measured by immunofluorescence. **(E)** *HOXA13* expression levels were examined in the above-mentioned paired PDAC tissues and their adjacent non-neoplastic tissues by PCR. **(F)** Correlation scatterplot (Spearman test) of HOTTIP and HOXA13 expression in PDAC (middle panel; B2) compared with para-tumor (right panel; B3) areas of the above tested tissues.

Because emerging evidence suggests that certain members of the *HOXA* cluster are involved in cancer progression, we hypothesized that HOTTIP might regulate the biological behavior of PDAC via regulation of the *HOXA* cluster. To confirm this hypothesis, we first evaluated the effect of HOTTIP knockdown on the expression of 5′ *HOXA* genes (*HOXA7*, *HOXA9*, *HOXA10*, *HOXA11*, *HOXA13*) by qRT-PCR. In SW1990 and MIA PaCa-2 cells, depletion of HOTTIP inhibited the expression of these genes to varying degrees, with the strongest inhibition observed for *HOXA13* (Figure 5A,B). Inhibition of HOXA13 levels was further confirmed by western blotting (Figure 5C). As expected, decreased nuclear immunofluorescence staining of HOXA13 was observed in SW1990 cells (Figure 5D). To confirm the expression and association between HOTTIP and HOXA13 expression levels, we evaluated the expression of HOXA13 in 90 paired resected samples, which included the eight PDAC samples used for the array analysis (Additional file 1: Figure S1A), and the presented panel of cell lines (Additional file 1: Figure S1B) by qRT-PCR. We observed enhanced expression of HOXA13 levels both in tumor tissues and cell lines (Figure 5E, Additional file 1: Figure S1B). Furthermore, we also observed a positive correlation between HOTTIP and HOXA13 expression levels both in cancer cell lines (Additional file 1: Figure S1C) and clinical samples (Figure 5F).

#### HOXA13 partly mediates the effect of HOTTIP on PDAC biology

**Figure 6:**
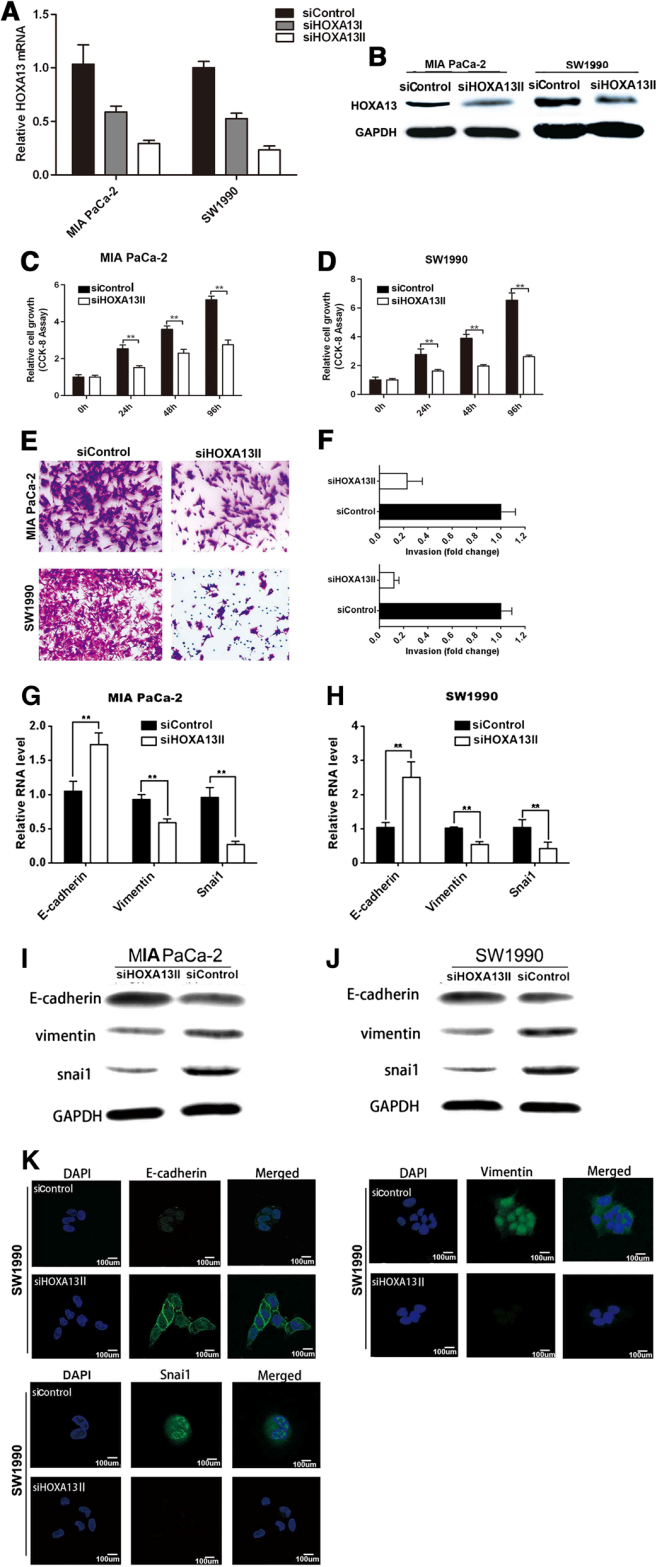
**HOXA13 partly mediates the effect of HOTTIP on PDAC biology. (A)** MIA PaCa-2 and SW1990 cells were transfected with control siRNA or siRNA against HOXA13, and *HOXA13* expression was subsequently determined by qRT­PCR. **(B)** HOXA13 knockdown efficiency was also confirmed by western blotting. **(C) (D)** Cell viability of MIA PaCa-2 **(C)** and SW1990 cells **(D)** was determined at the indicated time points by CCK-8 assays. **(E)**. Effect of HOXA13 on cell invasion ability was measured by Transwell assays. **(F)**. The number of invading cells was analyzed. **(G) (H)** The expression levels of EMT-related genes (*E-cadherin*, *Vimentin*, and *Snai1*) was evaluated by qRT-PCR 48 h after transfection in both cell lines. **(I) (J)** The effect of HOXA13 silencing on E-cadherin, Vimentin, and Snail 1 protein levels was confirmed by western blotting. **(K)** Immunofluorescence staining for the EMT makers in SW1990 cells. Data represent the mean ± s.d. from three independent experiments. **p < 0.01, Student’s t-test.

In the above section, we demonstrated that the strongest regulatory effect of HOTTIP among members of the *HOXA* cluster was on HOXA13 expression. To determine whether HOTTIP promotes PDAC progression by regulating HOXA13, we next employed siRNA to specifically silence the expression of HOXA13 in MIA PaCa-2 and SW1990 cells (Figure 6A,B). Strikingly, down-regulation of HOXA13 also inhibited PDAC cell growth (Figure 6C,D), invasion (Figure 6E,F), and EMT (Figure 6G-K). Taken together, these results suggest that the regulatory function of HOTTIP in PDAC biology acts, at least in part, by controlling HOXA13.

#### HOXA13 overexpression correlates with poor survival in PDAC

**Figure 7:**
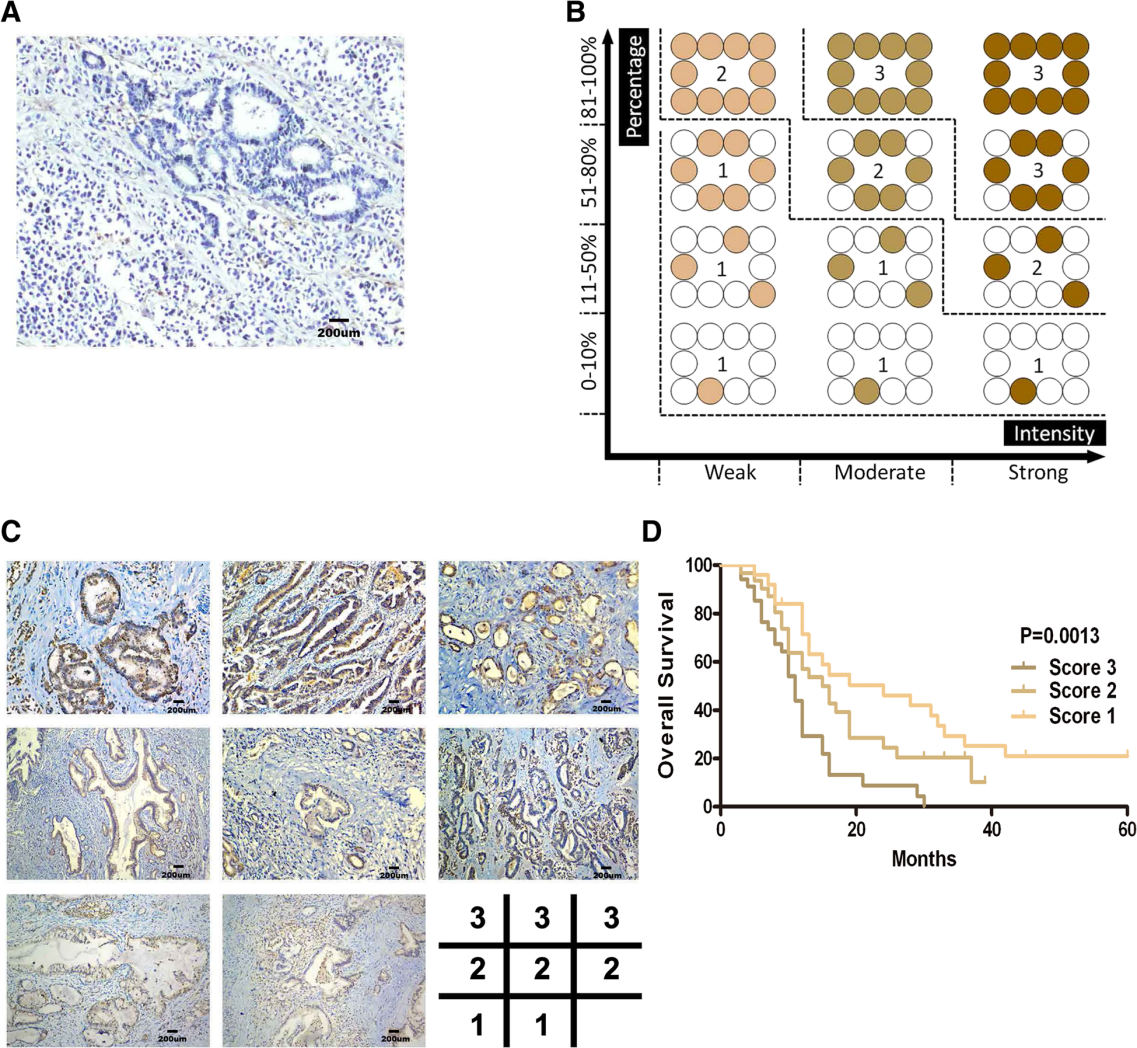
**HOXA13 overexpression correlates with poor survival in PDAC. (A)** A representative field of one PDAC specimen with a HOXA13 staining score of 0. **(B)** The scoring sketch for HOXA13 staining in PDAC tissues (score 1-score 3). **(C)** Representative images of HOXA13 staining in PDAC tissues (score 1-score 3). **(D)** Kaplan–Meier survival curves for patients with different HOXA13 staining scores in 90 cases of PDAC patients (The case got score 0 was classified into group of score 1; removing the case alone does not affect the statistical difference of survival analysis).

To determine the clinical relevance of the HOTTIP-HOXA13 axis in PDAC, levels of HOXA13 protein were measured in 90 paraffin-embedded, human PDAC samples by immunohistochemistry. As described in the Methods, the expression of HOXA13 was evaluated in terms of intensity and percentage separately, and finally expressed as a score of 0, 1, 2, or 3. In the present study, only one PDAC tissue specimen was designated a score of 0 (total negative staining) (Figure 7A). The scoring diagram from 1 to 3 is shown in Figure 7B, and representative images of score 1, score 2, and score 3 are shown in Figure 7C. We also analyzed the relationship between the intensity of HOXA13 staining and clinicopathologic features. Statistical analysis revealed that HOXA13 overexpression was correlated with lymph node metastasis and poor histological differentiation (Table 1). Furthermore, survival analysis revealed that higher HOXA13 staining intensity correlated with poorer prognosis in PDAC patients (Figure 7D).

**Table 1:** **Correlation of HOXA13 expression and clinicopathological characteristics**

**Factor**		**HOXA13 IHC scores**			P value*
		**1 (n=25)** ^**¶**^	**2 (n=31)**	**3 (n=34)**	
Age	<60	12	17	17	0.867
	≥60	13	14	17	
Sex	Male	15	20	22	0.920
	Female	10	11	12	
Differentiation	Well	15	12	6	0.0039
	Moderate	6	14	15	
	Poor	4	5	13	
UICC stage	pI	6	11	8	0.496
	pII	19	20	26	
T stage	T1	6	3	6	0.645
	T2	7	13	12	
	T3	12	15	16	
N stage	N0	16	12	9	0.014
	N1	9	19	25	
Perineural invasion	Negative	11	16	19	0.664
	Positive	14	15	15	

^¶^For convenience, the case get score 0 was included in the group of score 1; removing the case of score 0 alone does not affect the statistical differences in any event.

*Pearson Chi-Square test.

## Discussion

The prognosis of PDAC is extremely poor, therefore, understanding the mechanisms underlying PDAC pathogenesis may help yield novel biomarkers for early detection and treatment [8]. Recent studies have shown that dysregulated expression of lncRNAs in solid cancers reflect disease progression and may independently predict patient outcome [22,23]. In the present study, we demonstrate that a lncRNA HOXA transcript at the distal tip (HOTTIP, is associated with PDAC tumor progression and disease outcome.

Rinn and coworkers have identified up to 3000 lncRNAs [28,29], and biological characterization of these demonstrates that lncRNAs are master regulators of embryonic pluripotency, differentiation, and body axis patterning. In addition, lncRNAs have been shown to promote developmental transitions and regulate histone modifications and hence influence the epigenetic programs of the transcriptome [30,31]. Of note, lncRNAs may also play roles as drivers of tumor suppression or exert oncogenic functions in a wide variety of cancer types, by sustaining tumor cell proliferation, evading growth suppressors, enabling replicative immortality, inducing angiogenesis, and promoting invasion and metastasis [32-34].

To date, many transcribed lncRNAs have been implicated in gene silencing [35,36], but the potential roles of lncRNAs in gene activation are much less understood [28,37]. Recently, a lncRNA named HOTTIP, which resides at the 5′ tip of the *HOXA* locus and coordinates the activation of multiple 5′ *HOXA* genes *in vivo* [24], has been identified as one of 231 lncRNAs associated with the human *HOX* loci [28]. This lncRNA directly binds the adaptor protein WDR5 and targets WDR5/MLL complexes across *HOXA*, thus driving histone H3 lysine 4 trimethylation and gene transcription [24]. Furthermore, expression of HOTTIP has been identified as a negative prognostic factor in hepatocellular carcinoma patients [25]; however, the functional role of HOTTIP in cancer progression remains unknown. This present study offers the first insight into the effect of HOTTIP on malignant cell behavior. Our results demonstrate that HOTTIP, which is overexpressed in human pancreatic cancer tissues and cells compared with non-tumoral tissues and cell lines, enhances pancreatic cell proliferation and invasion as well as EMT. In addition, we show that targeted silencing of HOTTIP potentiates the antitumor effects of gemcitabine both *in vitro* and *in vivo*.

The *HOX* family of homeobox genes encodes transcriptional regulators that are expressed during development in regionalized domains along the main body axis (limb, lung, gut, and lower genitourinary tract), where they regulate cell proliferation and differentiation [38]. Additionally, *HOX* genes represent the most repeat-poor regions within the human genome and display a unique gene network organization (four chromosomal loci: A, B, C, and D). The *HOXA* locus consists of a cluster of 11 *HOX* genes with a graded expression pattern along body appendages from proximal (close to the main body) to distal (appendage tip) [39,40]. Among the *HOXA* genes, *HOXA13*, which is a marker of gut primordial posteriorization during development [41], has been shown to play a crucial role in tumorigenesis of the liver and bladder and in esophageal cancer [42-44]. In the present study, *HOXA13* was the most significantly inhibited gene within the *HOXA* locus following depletion of HOTTIP in pancreatic cancer cells. Located in physical continuity (chr7p 15.2) with the *HOXA13* gene [24], HOTTIP is expressed from development to adulthood in lumbosacral anatomc regions, explaining this regulatory relationship. Our observation is also in agreement with previously published data conducted in fibroblasts from a distal anatomic site (foreskin) [24]. Moreover, we found that HOTTIP and HOXA13 expression was strongly positively correlated in 90 PDAC tissues and in their corresponding adjacent nonneoplastic tissues. In addition, siRNA-mediated HOXA13-knockdown inhibited the proliferation, invasion, and EMT of PDAC cells, which was consistent with the functional changes that occurred after silencing the expression of HOTTIP in PDAC cells. In addition to the HOTTIP-targeted regulation of HOXA13 expression, we also observed reduced HOTTIP levels upon siRNA-mediated knockdown of HOXA13 in two different pancreatic cancer cell lines. Targeted inhibition of HOXA13 in SW1990 cells led to a 77% reduction in the mRNA level of HOXA13, and HOTTIP expression was also reduced by 48.2%. Similarly, in MIA PaCa-2 cells, HOXA13 levels were reduced to 29.3%, with a concomitant 39.6% reduction in HOTTIP levels. Our data are consistent with a pioneering study demonstrating that decreased expression of HOXA13 led to a clear reduction of HOTTIP expression in liver cancer-derived cell lines [26]. Thus, our work confirms that the regulatory loop between HOTTIP and its target, HOXA13, is also preserved during pancreatic cancer tumorigenesis. Taken together, our results support a mechanism whereby the HOTTIP/HOXA13 axis plays a critical role in PDAC tumorigenesis. Analysis of the relationship between HOXA13 expression in paraffin-embedded PDAC samples and clinicopathological data indicates that patients with high HOXA13 expression exhibit increased lymph node metastasis, poor histological differentiation, and decreased overall survival (Table 1, Figure 5). Hence, our findings are clinically and functionally relevant to the progression of human PDAC.

## Conclusions

Overall, our present study demonstrates that HOTTIP, which is significantly overexpressed in PDAC, plays a significant role in PDAC progression and gemcitabine chemoresistance. Our results also show that HOTTIP exerts its function in PDAC at least partly by controlling HOXA13. Further studies are required to validate the molecular axis involving HOTTIP and HOXA13 as a predictive biomarker, as well as a therapeutic target in PDAC. A deeper understanding of the function and downstream signaling pathways influenced by HOTTIP/HOXA13 deregulation may provide novel insights into the mechanisms underlying PDAC tumorigenesis.

## Additional file

Additional file 1: Figure S1. (A). Correlation scatterplot (Spearman test) of HOTTIP and HOXA13 expression in the eight PDAC samples used for microarray compared with their paired para-tumor tissues.(B). HOXA13 expression was evaluated in five pancreatic cancer cell lines compared with HPDE6 cells via qRT-PCR. mRNA levels were normalized to *β-actin*. HOTTIP levels in these cell lines are also shown (the same data as described in Figure 1C). (C). The correlation between *HOTTIP* and *HOXA13* mRNA expression in five pancreatic cancer cell lines was evaluated using Spearman correlation analysis.

## Abbreviations

PC: Pancreatic cancer
PDAC: Pancreatic ductal adenocarcinoma
HOTTIP: HOXA transcript at the distal tip
EMT: Epithelial mesenchymal transition
lncRNA: Long non-coding RNA
