# Supplementary figures and images for: The long non-coding RNA HOTTIP promotes progression and gemcitabine resistance by regulating HOXA13 in pancreatic cancer
Source: J Transl Med. 2015 Mar 12;13:84. doi: 10.1186/s12967-015-0442-z (PMC4372045; doi:10.1186/s12967-015-0442-z)

A

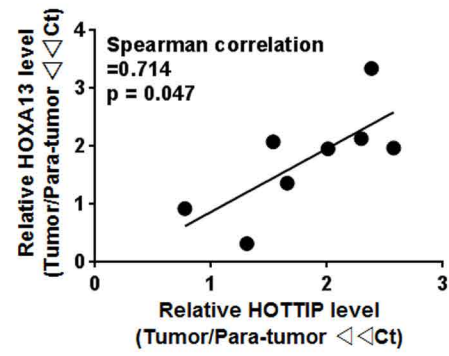

B

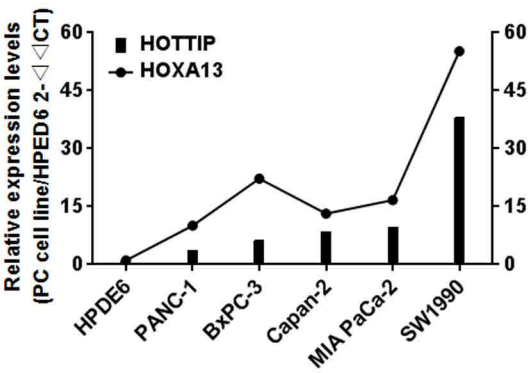

C

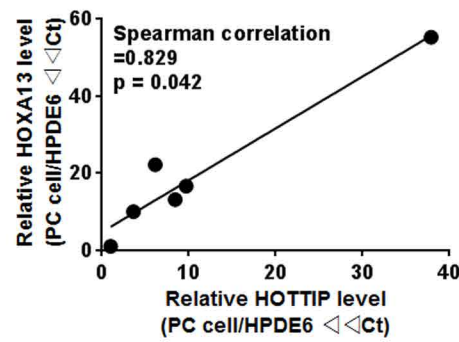

Supplement: Additional file 1: Figure S1. — (A). Correlation scatterplot (Spearman test) of HOTTIP and HOXA13 expression in the eight PDAC samples used for microarray compared with their paired para-tumor tissues.(B). HOXA13 expression was evaluated in five pancreatic cancer cell lines compared with HPDE6 cells via qRT-PCR. mRNA levels were normalized to β-actin. HOTTIP levels in these cell lines are also shown (the same data as described in Figure 1C). (C). The correlation between HOTTIP and HOXA13 mRNA expression in five pancreatic cancer cell lines was evaluated using Spearman correlation analysis. [file 12967_2015_442_MOESM1_ESM.pdf]
